# Safety and Tolerability of Oral Islatravir Once Monthly as Pre-exposure Prophylaxis in Cisgender Men and Transgender Women Who Have an Elevated Likelihood of HIV-1 Exposure: Results From the IMPOWER-24 Randomized Phase 3 Study

**DOI:** 10.1093/cid/ciag171

**Published:** 2026-03-14

**Authors:** Raphael J Landovitz, Yvett Pinedo, Federico Hinestrosa, Gordon E Crofoot, Cynthia Brinson, Susan Buchbinder, Jean-Michel Molina, Ronnie M Gravett, James B Brock, Moti N Ramgopal, A Lina Rosengren, Igho Ofotokun, Jose Valdez Madruga, Geoffroy Liegeon, Ravindre Panchia, Khuanchai Supparatpinyo, Anchalee Avihingsanon, Catherine Creticos, Shobha Swaminathan, Susanne Doblecki-Lewis, Jorge Rodriguez, Marc Siegel, Shinichi Oka, Thanyawee Puthanakit, Beatriz Grinsztejn, Eduard J Sanders, Javier R Lama, Johannes Lombaard, Nkosiphile Ndlovu, Peggy Hwang, Jiejun Du, Beth Jackson, Brenda Homony, Barbara Evans, Peter Sklar, Michael N Robertson, Rebeca M Plank

**Affiliations:** Department of Medicine, UCLA Center for Clinical AIDS Research and Education, Los Angeles, California, USA; Clinical Research Unit, Via Libre, Lima, Peru; Department of Infectious Diseases, Orlando Immunology Center, Orlando, Florida, USA; The Crofoot Research Center, Houston, Texas, USA; Central Texas Clinical Research, Austin, Texas, USA; Population Health Division, San Francisco Department of Health, San Francisco, California, USA; Department of Infectious Diseases, Paris Cité University, Paris, France; Assistance Publique, Hôpitaux de Paris, Hôpitaux Saint Louis et Lariboisière, Paris, France; INSERM UMR1342, Paris, France; Division of Infectious Diseases, University of Alabama at Birmingham, Birmingham, Alabama, USA; Department of Medicine, Division of Infectious Diseases, University of Mississippi Medical Center, Jackson, Mississippi, USA; Midway Immunology and Research Center, Fort Pierce, Florida, USA; Division of Infectious Diseases, The University of North Carolina at Chapel Hill, Chapel Hill, North Carolina, USA; Department of Medicine, Emory University School of Medicine, Atlanta, Georgia, USA; Centro de Referência e Treinamento DST/AIDS-SP, São Paulo, Brazil; Department of Infectious Diseases, Paris Cité University, Paris, France; Assitance Publique—Hôpitaux de Paris, Hôpitaux Saint Louis et Lariboisière, Paris, France; INSERM UMR 1137 IAME—Team PreViST, Paris, France; School of Public Health, Perinatal HIV Research Unit (PHRU)-HIV Prevention CRS, University of the Witwatersrand, Johannesburg, South Africa; Research Institute for Health Sciences, Chiang Mai University, Chiang Mai, Thailand; HIV-NAT, Thai Red Cross AIDS and Infectious Diseases Research Centre and Center of Excellence in Tuberculosis, Faculty of Medicine, Chulalongkorn University, Bangkok, Thailand; Howard Brown Health Center, Chicago, Illinois, USA; Division of Infectious Diseases, Department of Medicine, Rutgers New Jersey Medical School, Newark, New Jersey, USA; Division of Infectious Diseases, University of Miami Miller School of Medicine, Miami, Florida, USA; Global Research Institute, Los Angeles, California, USA; School of Medicine and Health Sciences, George Washington University, Washington DC, USA; AIDS Clinical Center, National Center for Global Health and Medicine, Tokyo, Japan; Department of Pediatrics, Faculty of Medicine, Chulalongkorn University, Bangkok, Thailand; Evandro Chagas National Institute of Infectious Diseases-Fiocruz, Rio de Janeiro, Brazil; Implementation Research Division, Aurum Institute, Johannesburg, South Africa; Department of Research, Asociación Civil Impacta Salud y Educación, Lima, Peru; Josha Research, Bloemfontein, South Africa; Wits RHI, Faculty of Health Sciences, University of the Witwatersrand, Johannesburg, South Africa; MRL, Merck & Co., Inc., Rahway, New Jersey, USA; MRL, Merck & Co., Inc., Rahway, New Jersey, USA; MRL, Merck & Co., Inc., Rahway, New Jersey, USA; MRL, Merck & Co., Inc., Rahway, New Jersey, USA; MRL, Merck & Co., Inc., Rahway, New Jersey, USA; MRL, Merck & Co., Inc., Rahway, New Jersey, USA; MRL, Merck & Co., Inc., Rahway, New Jersey, USA; MRL, Merck & Co., Inc., Rahway, New Jersey, USA

**Keywords:** islatravir, PrEP, HIV-1 prevention, cisgender men, transgender women

## Abstract

**Background:**

Islatravir once monthly (qm), a nucleoside reverse transcriptase translocation inhibitor with a long half-life, was evaluated for safety and tolerability in cisgender men and transgender women who have sex with men and are at increased likelihood of HIV-1 (HIV) exposure.

**Methods:**

IMPOWER-24 (NCT04652700) was a double-blind, Phase 3 study. Participants were randomized 2:1 to islatravir 60 mg oral qm or emtricitabine (FTC; 200 mg) coformulated with either tenofovir disoproxil (245 mg) or tenofovir alafenamide (TAF; 25 mg) once daily (qd). After ∼9 months, blinded islatravir was discontinued due to lymphocyte reductions; participants were offered open-label comparator for 20 months.

**Results:**

In total, 494 participants were enrolled (328 islatravir; 166 comparator): 91.5% were cisgender men, 41.7% were White, and median age was 27 years. Mean blinded dosing duration was 4.7 months (islatravir) versus 4.3 months (comparator). Overall, 211 participants (64.3%) in the islatravir group and 128 (77.1%) in the comparator group had ≥1 adverse event (AE). Most AEs were mild or moderate, with 1 AE leading to product discontinuation (islatravir; gastroesophageal reflux). Serious AEs occurred in <2%; none were related to study product. Change in total lymphocytes in the islatravir group at Month 3 was −7.4%; a trend toward recovery was observed after islatravir was stopped. Mean total lymphocytes remained within normal range. No HIV infections occurred in either group during the double-blind phase.

**Conclusions:**

Islatravir qm was generally well tolerated; decreases in total lymphocytes were observed with islatravir. Original primary efficacy objectives were not assessed due to early study stoppage.

## Teaser

Islatravir 60 mg oral qm for PrEP in cisgender men and transgender women at increased likelihood of HIV exposure demonstrated an AE profile generally similar to comparator. Islatravir recipients experienced lymphocyte decreases, which were not associated with increased infection rates.

## BACKGROUND

Significant progress has been made in reducing new cases of HIV-1 (referenced as HIV hereafter) and HIV-2, with new infections declining to an estimated 1.3 million in 2023, representing a 39% reduction since 2010 and a 60% reduction since 1995; however, this is still below the target of fewer than 370 000 new HIV and HIV-2 infections globally by 2025 [1, 2]. Progress is further threatened by limitations in domestic and global support for HIV and HIV-2 prevention and treatment services [3]. Among the global general population (aged 15–49 years), the prevalence of HIV and HIV-2 is disproportionately higher (7.7%) in cisgender men and transgender women who have sex with men [2]. In 2015, the World Health Organization (WHO) adopted the recommendation of offering pre-exposure prophylaxis (PrEP) to all people with an elevated likelihood of HIV exposure, which led to inclusion in national policies and programs [4]. Although more than 3.5 million people received PrEP in 2023, use is still below the target of 10 million people using PrEP by 2025 [1]. In 2012, the US Food and Drug Administration approved emtricitabine/tenofovir disoproxil (FTC/tenofovir disoproxil) taken orally once daily (qd) as PrEP, for prevention of HIV acquisition; FTC with tenofovir alafenamide (TAF) was approved as PrEP for men who have sex with men and transgender women in 2019 [5, 6]. Currently approved PrEP regimens include daily oral regimens, a long-acting vaginal ring (with European Medicines Agency approval for use in countries with high disease burden), and long-acting injectable options; all of these formulations present various challenges [7–10], and oral long-acting PrEP could provide an additional choice to overcome existing barriers to PrEP uptake, even given the demonstrated success of currently available PrEP agents [11].

Islatravir is a nucleoside reverse transcriptase translocation inhibitor (NRTTI), which was evaluated as a long-acting oral PrEP and is still under investigation for treatment of HIV in combination with other antiretrovirals [12, 13]. The active metabolite islatravir triphosphate (islatravir-TP) has nanomolar potency against wild-type HIV, as well as against variants resistant to nucleos(t)ide reverse transcriptase inhibitors (NRTIs) (eg, M184I/V), and has a terminal half-life in human peripheral blood mononuclear cells (PBMCs) of 195 hours [13–15]. In Phase 1 studies in adults without HIV, single doses (5–400 mg) and multiple doses (10–100 mg once weekly [qw] for 3 weeks) of islatravir were generally well tolerated [12]. In a Phase 2 study in adults with low likelihood of HIV exposure, islatravir 60 and 120 mg once monthly (qm) were generally well tolerated and achieved the prespecified pharmacokinetics (PK) threshold for PrEP [16]. Although islatravir is not being further developed as oral or implantable PrEP due to the reasons outlined in the following paragraph, the findings from this study will inform future development of other long-acting agents for HIV prevention, including MK-8527, a novel NRTTI currently under clinical development as an oral qm PrEP option [17–20].

MK-8591-024 (IMPOWER-24) was a randomized, active-controlled, parallel-group, double-blind, double-dummy, Phase 3 study that was originally designed to investigate the efficacy of islatravir oral qm for reducing HIV infection relative to the counterfactual background rate and to study the safety and tolerability of islatravir qm compared with FTC/tenofovir disoproxil or FTC/TAF oral qd in cisgender men and transgender women who have sex with men. However, due to dose-dependent decreases in total lymphocyte and CD4+ T-cell counts observed across the islatravir development program [21], enrollment and blinded intervention were prematurely stopped. Therefore, prespecified safety and efficacy endpoints could not be formally evaluated. Here, we summarize the available data, including safety and tolerability, which became the amended primary objectives of the study.

## METHODS

### Study Design and Participants

This multicenter, Phase 3 study (protocol MK-8591-024; NCT04652700) was conducted to evaluate oral islatravir in cisgender men and transgender women (≥16 years) who have sex with men with an increased likelihood of being exposed to HIV. Approximately 1500 participants were planned for inclusion. Participants were assessed as HIV uninfected for eligibility purposes based on a negative HIV-1/HIV-2 test performed at screening and on Day 1. Participants taking FTC/tenofovir disoproxil or FTC/TAF (US cohort only) as PrEP at screening were allowed to continue until the day of randomization. Exclusion criteria included evidence of chronic HBV (HBsAg positive), or past HBV (HBsAg negative and HBcAb positive) infection that indicated risk for hepatitis B reactivation, current or chronic history of liver disease, and prior or current use of any other long-acting HIV prevention product.

### Study Objectives, Intervention, and Procedures

The primary objectives of this study were to evaluate the efficacy of islatravir oral qm in reducing the incidence of HIV infection relative to the counterfactual background rate and to evaluate the safety and tolerability of islatravir oral qm compared with FTC/tenofovir disoproxil or FTC/TAF qd. Enrollment and the blinded phase of the study were stopped early after about 9 months (study start date, 15 March 2021; end of blinded intervention, 10 December 2021). As a result, the protocol was amended, and efficacy was no longer considered a primary objective of the study; the amended primary study objective was to evaluate the safety and tolerability of islatravir qm. The primary safety outcomes were the incidence of adverse events (AEs) overall and the incidence of AEs leading to discontinuation of study intervention. Additional safety measures included other AE categories (eg, related to study intervention, toxic event Grade 3 or 4, and serious AEs) and events of clinical interest (eg, changes in total lymphocyte counts).

**Figure 1.**
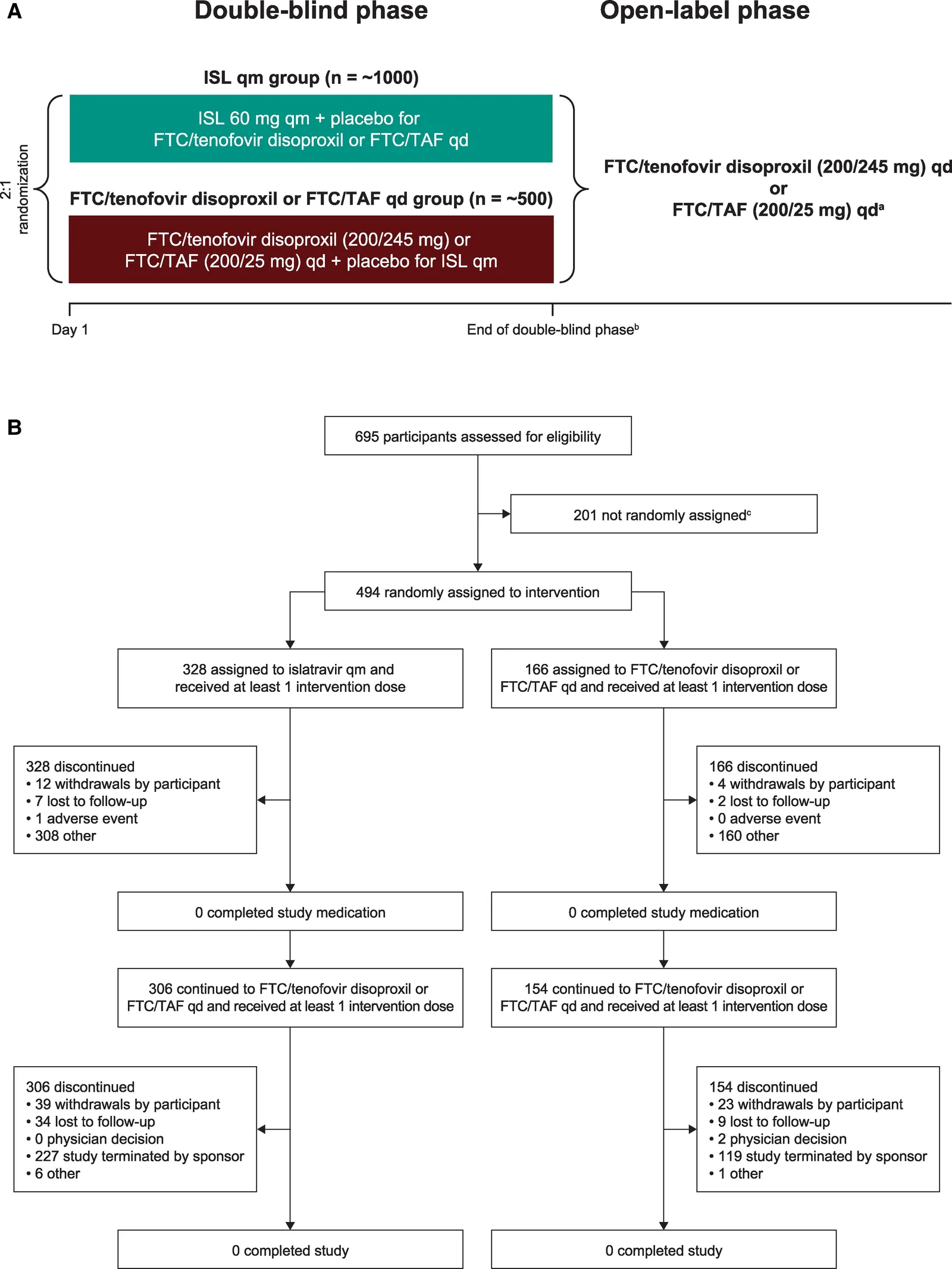
IMPOWER-24. *A*, Planned study design and (*B*) participant disposition. Abbreviations: FTC, emtricitabine; ISL, islatravir; qd, once daily; qm, once monthly; TAF, tenofovir alafenamide. ^a^Each FTC/tenofovir disoproxil tablet contained FTC 200 mg and tenofovir disoproxil 245 mg (equivalent to tenofovir disoproxil fumarate 300 mg or tenofovir disoproxil phosphate 291.22 mg). FTC/TAF was used in the US cohort only. ^b^End of double-blind phase included a safety follow-up period of 42 d after the last dose of study intervention. ^c^Among individuals who were screened but not randomly assigned (n = 201), 93 did not meet inclusion criteria or met the exclusion criteria; 22 of 93 individuals (23.7%) had at least 1 positive HIV test result at screening.

After a screening period of ≤45 days, eligible participants were randomly assigned 2:1 to the islatravir qm group (islatravir 60 mg oral qm + placebo for FTC/tenofovir disoproxil or FTC/TAF qd) or the active comparator group (FTC/tenofovir disoproxil 200/245 mg [tenofovir disoproxil 245 mg was equivalent to tenofovir disoproxil fumarate 300 mg or tenofovir disoproxil phosphate 291.22 mg] or FTC/TAF 200/25 mg oral qd + placebo for islatravir qm) (double-blind phase; Figure 1*A*). The randomized allocation schedule for study intervention assignment was generated by the sponsor's clinical biostatistics department, and randomization was implemented using an interactive response technology. Randomization was stratified by geographic region/race (ie, United States [Black/African American], United States [non-Black/non–African American], Africa, and other). Islatravir and FTC/tenofovir disoproxil were packaged identically to their matching placebos to conceal the intervention assignment from the participant, the investigator, and sponsor personnel or staff who were involved in the administration of study intervention or the clinical evaluation of participants. The study was unblinded when the data were declared final and complete. The double-blind phase included a safety follow-up period of 42 days after the last dose of study intervention (based on the terminal half-life of islatravir measured in PBMCs of 195 hours [14]). After discontinuation of the randomized blinded phase, all participants were offered open-label FTC/tenofovir disoproxil (200/245 mg) qd or FTC/TAF (200/25 mg) qd for HIV PrEP while continuing in the study for safety monitoring (open-label phase; Figure 1*A*). Although enrollment of participants aged 16–17 years had been planned, no participant <18 years of age was enrolled before study closure.

Blood samples were collected from all participants for laboratory tests, which included a hematology test panel, chemistry test panel, viral hepatitis serology screening, sexually transmitted infection (STI) screening, and biomarker testing (for genetic analysis and future biomarker research). Sexually transmitted infection testing was conducted and reported in aggregate as follows: (1) positive results from mandatory protocol-based testing, including cases where participant was asymptomatic; and (2) participants presenting with symptoms or clinical signs of STIs were tested, with results recorded as symptomatic or investigator-reported AEs. Blood samples were also used to confirm HIV status by point-of-care testing, antibody/antigen testing, real-time polymerase chain reaction (to confirm HIV infection), and HIV drug resistance testing (performed only when viremia [HIV RNA ≥ 200 copies/mL] was detected). Adverse events were collected at each study visit and were graded according to the Division of AIDS (DAIDS; version 2.1) criteria [22]. A complete physical examination was performed at screening and symptom-directed physical examinations at all subsequent visits (monthly). Vital signs were measured during each visit and included weight, temperature, pulse, respiratory rate, and systolic and diastolic pressure. Any clinically meaningful abnormalities in physical examinations, as assessed by the investigator or qualified site staff, or vital signs were reported as AEs. The original study protocol was amended on 6 December 2021 to include blood samples at additional time points for measuring total lymphocyte, CD4+ T-cell, and CD8+ T-cell counts.

### Statistical Analysis

As a result of early stopping of the study, formal hypothesis tests and comparisons between study intervention groups were not conducted. This report describes the safety and tolerability of islatravir qm based on review of the accumulated safety data. No prespecified statistical analyses were planned for AEs, and the study was not powered to evaluate safety outcomes statistically.

Adverse event data were evaluated for the blinded treatment period, including a follow-up period of 42 days after the last dose of blinded study intervention. Lymphocyte data were evaluated for the entire duration of the study to assess the recovery of total lymphocyte counts. The mean percent change from baseline in total lymphocyte count was calculated from all available data at each study visit, and the within-group 95% CIs were calculated based on the *t* distribution.

The number of participants who tested positive for HIV infection (as confirmed by a blinded clinical adjudication committee) was assessed in the modified full-analysis set, which included participants who received ≥1 dose of study intervention and did not have confirmed HIV infection before or at randomization.

## RESULTS

### Study Participants

Participants were assigned at 23 sites in France, Japan, Peru, South Africa, Thailand, and the United States (Supplementary Table 1). A total of 695 participants were screened, and 494 participants were randomly assigned: 328 to the islatravir qm group and 166 to the FTC/tenofovir disoproxil or FTC/TAF qd group. Mean duration of dosing was 4.7 months (range, 1–10 months) in the islatravir qm group and 130.1 days/4.3 months (range, 1–282 days/1–9.3 months) in the FTC/tenofovir disoproxil or FTC/TAF qd group (Table 1). After the early stopping of the double-blind phase of the study, 93.1% of participants entered the open-label phase: 306 (93.3%) from the islatravir qm group and 154 (92.8%) from the FTC/tenofovir disoproxil or FTC/TAF qd group (Figure 1*B*).

**Table 1.** Duration of Dosing for Islatravir qm and FTC/Tenofovir Disoproxil or FTC/TAF qd Groups Until the Blinded Phase of the Study Was Discontinued^a^

Islatravir qm		FTC/Tenofovir Disoproxil or FTC/TAF qd	
Months	n	Weeks	n
1	50	≤4	15
2–3	110	>4–12	60
4–6	43	>12–36	84
7–10	125	>36	7

Abbreviations: FTC, emtricitabine; qd, once daily; qm, once monthly; TAF, tenofovir alafenamide.

^a^The observed differences in participant counts reflect study design and follow-up duration.

The baseline characteristics and demographics were comparable between the intervention groups. Overall, 91.5% of the participants identified as cisgender men and 4.5% as transgender women. The study population had a median age of 27 (range, 18–76) years. Most of the participants were White (41.7%), followed by Black or African American (25.1%) and Asian (20.4%). Additionally, 32.8% of the participants were of Hispanic or Latine ethnicity, and 44.1% had no previous history of daily oral PrEP use (Table 2).

**Table 2.** Demographics and Baseline Characteristics

n (%)	Total, N = 494	Islatravir qm, n = 328	FTC/Tenofovir Disoproxil or FTC/TAF qd, n = 166
Gender identity			
Cisgender male	452 (91.5)	301 (91.8)	151 (91.0)
Transgender female	22 (4.5)	14 (4.3)	8 (4.8)
GQ/NB/GNC AMAB	20 (4.0)	13 (4.0)	7 (4.2)
Age			
18 to <30 y	335 (67.8)	221 (67.4)	114 (68.7)
≥30 y	159 (32.2)	107 (32.6)	52 (31.3)
Median (range), years	27 (18–76)	27 (18–76)	27 (18–65)
Race			
White	206 (41.7)	135 (41.2)	71 (42.8)
Black/African American	124 (25.1)	83 (25.3)	41 (24.7)
Asian	101 (20.4)	70 (21.3)	31 (18.7)
Other^a^	63 (12.8)	40 (12.2)	23 (13.9)
Hispanic or Latine	162 (32.8)	111 (33.8)	51 (30.7)
Country			
France	12 (2.4)	6 (1.8)	6 (3.6)
Japan	26 (5.3)	20 (6.1)	6 (3.6)
Peru	52 (10.5)	33 (10.1)	19 (11.4)
South Africa	59 (11.9)	39 (11.9)	20 (12.0)
Thailand	68 (13.8)	46 (14.0)	22 (13.2)
United States	277 (56.1)	184 (56.1)	93 (56.0)
Prior use of daily oral PrEP^b^	276 (55.9)	185 (56.4)	91 (54.8)
Recent prior user of daily oral PrEP^c^	249 (50.4)	166 (50.6)	83 (50.0)

Abbreviations: FTC, emtricitabine; GQ/NB/GNC AMAB, genderqueer/nonbinary/gender nonconforming assigned male at birth; PrEP, pre-exposure prophylaxis; qd, once daily; qm, once monthly; TAF, tenofovir alafenamide.

^a^Includes American Indian or Alaska Native, multiple/multiracial, Native Hawaiian or other Pacific Islander, and race unknown.

^b^A participant was considered a prior user of PrEP if there was a record of PrEP use before the start of the study.

^c^A participant was considered a recent prior user of PrEP if there was a record of PrEP use within 90 days of screening.

### Safety

#### Adverse Events

The proportion of participants with ≥1 AEs was comparable between the intervention groups during the double-blind phase and the safety follow-up period of 42 days after the last dose of blinded study intervention: 211 (64.3%) in the islatravir qm group and 128 (77.1%) in the FTC/tenofovir disoproxil or FTC/TAF qd group (Table 3). The proportion of participants with drug-related AEs was also similar between the groups: 33 (10.1%) in the islatravir qm group and 16 (9.6%) in the FTC/tenofovir disoproxil or FTC/TAF qd group. Serious AEs occurred in <2% of participants in each group. Most AEs were considered mild or moderate (Grade 1 or 2); <3% of participants had Grade 3 or 4 AEs. Only 1 Grade 3 AE (postacute coronavirus disease 2019 [COVID-19] syndrome in the islatravir qm group) was considered related to study intervention by the investigator.

**Table 3.** Adverse Events and Most Common Infections During the Double-Blinded Phase^a^

Participants, n (%)	Islatravir qm, n = 328	FTC/Tenofovir Disoproxil or FTC/TAF qd, n = 166
≥1 AE	211 (64.3)	128 (77.1)
≥1 drug-related AE	33 (10.1)	16 (9.6)
Grade 3 or 4 AEs^b^	9 (2.7)	3 (1.8)
Serious AEs	6 (1.8)	1 (0.6)
Discontinuations due to AEs	1 (0.3)	0
Infections (≥1), n (%)	122 (37.2)	83 (50.0)
Most common infections (≥1) in any study group, n (%)		
COVID-19	36 (11.0)	22 (13.3)
Chlamydial proctitis	20 (6.1)	14 (8.4)
Pharyngitis	3 (0.9)	3 (1.8)
Nasopharyngitis	14 (4.3)	7 (4.2)
Gonococcal proctitis	13 (4.0)	15 (9.0)
Upper respiratory tract infection	10 (3.0)	8 (4.8)
Syphilis	10 (3.0)	7 (4.2)
Influenza	4 (1.2)	5 (3.0)
Tonsillitis	4 (1.2)	1 (0.6)
Urethritis gonococcal	4 (1.2)	1 (0.6)
Pharyngitis streptococcal	1 (0.3)	3 (1.8)
Bronchitis	3 (0.9)	2 (1.2)
Folliculitis	3 (0.9)	2 (1.2)
Gastroenteritis	2 (0.6)	3 (1.8)
Sinusitis	1 (0.3)	3 (1.8)
Syphilis	1 (0.3)	3 (1.8)
Cellulitis	1 (0.3)	2 (1.2)
Genitourinary chlamydia infection	1 (0.3)	2 (1.2)
Gonorrhea	0	3 (1.8)
Chlamydial infection	0	2 (1.2)

Abbreviations: AE, adverse event; COVID-19, coronavirus disease 2019; DAIDS, Division of AIDS; FTC, emtricitabine; qd, once daily; qm, once monthly; TAF, tenofovir alafenamide.

^a^Data collected during the double-blind phase and through the safety follow-up of 42 days after the last dose of blinded study intervention.

^b^DAIDS (version 2.1) table for the grading of the severity of adult and pediatric AEs [22].

Six participants (1.8%) in the islatravir qm group had serious AEs: proctitis, appendicitis, COVID-19, folliculitis, pneumonia, hyperglycemia, and worsening of epilepsy (which did not require treatment modification); none of these events were considered related to the study intervention. One participant discontinued treatment from the islatravir qm group due to a Grade 2 AE of gastroesophageal reflux disease, which was considered related to the study intervention by the investigator. There were no deaths reported during the study.

The most common AE (≥10%) in either group was COVID-19 (islatravir qm, 36 [11.0%]; FTC/tenofovir disoproxil or FTC/TAF qd, 22 [13.3%]; Table 3). Other frequently reported AEs (>5%) were chlamydial proctitis (6.1%) and diarrhea (5.8%) in the islatravir qm group and gonococcal proctitis (9.0%), chlamydial proctitis (8.4%), oropharyngeal gonococcal infection (6.6%), headache (6.0%), and diarrhea (5.4%) in the FTC/tenofovir disoproxil or FTC/TAF qd group. The most frequently reported class of AEs was infections and infestations, which occurred in 37.2% of the islatravir qm group and 50.0% of the FTC/tenofovir disoproxil or FTC/TAF qd group. The incidence of specific infections was generally similar between the intervention groups during the double-blind phase of the study (Table 3) and when all available data were analyzed (data obtained during the double-blind and open-label phase; Supplementary Table 2).

#### Change in Total Lymphocyte Counts

**Figure 2.**
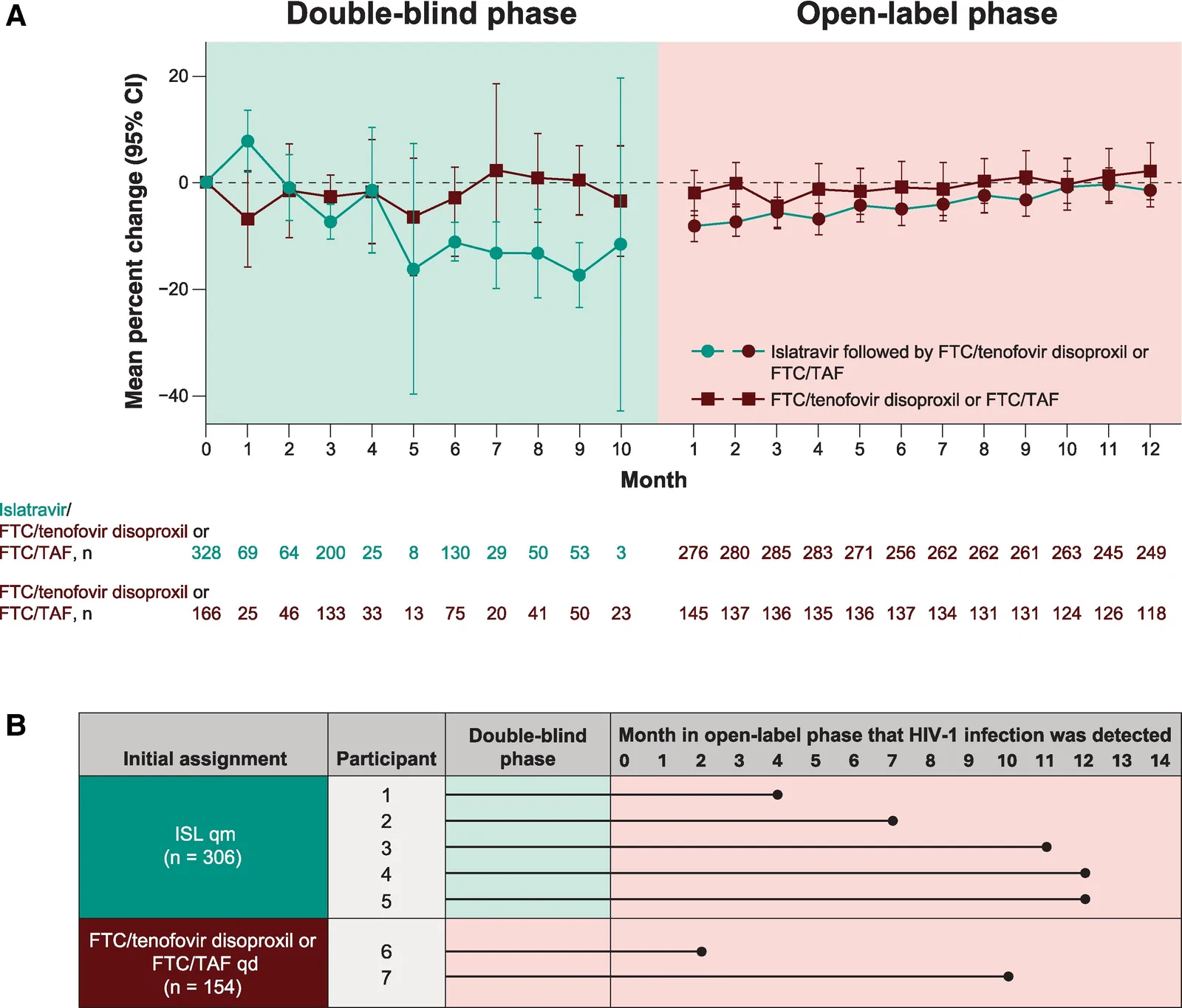
*A*, Mean percent change from baseline in total lymphocytes and (*B*) HIV acquisition during the open-label phase. Abbreviations: FTC, emtricitabine; ISL, islatravir; qd, once daily; qm, once monthly; TAF, tenofovir alafenamide.

Decreased total lymphocyte counts were observed with islatravir qm in this study. At double-blind Month 3, the mean percent change from baseline in total lymphocyte count was −7.4% (95% CI, −10.6 to −4.1; n = 200) in the islatravir qm group and −2.7% (95% CI, −6.9–1.4; n = 133) in the FTC/tenofovir disoproxil or FTC/TAF qd group. At double-blind Month 9, the mean percent change in total lymphocyte count was −17.4% (95% CI, −23.4 to −11.4; n = 53) in the islatravir qm group versus 0.4% (95% CI, −6.1–6.9; n = 50) in the FTC/tenofovir qd group. A trend toward recovery was observed after islatravir qm administration was stopped. At open-label Month 12, the mean percent change from baseline in total lymphocyte count was −1.5% (95% CI, −4.6–1.6; n = 249) for the islatravir qm group and 2.1% (95% CI, −3.2–7.4; n = 118) for the FTC/tenofovir disoproxil or FTC/TAF qd group (Figure 2*A*).

Two participants in the islatravir qm group experienced a DAIDS Grade 3 (<500 cells/mm^3^) decrease in lymphocyte counts. No participants had CD4+ T-cell counts < 200 cells/mm^3^ during the double-blind study intervention or within 42 days following discontinuation.

### HIV Acquisition

No participants acquired HIV during the double-blind phase or 42 days after discontinuation of islatravir qm. In the open-label phase with FTC/tenofovir disoproxil or FTC/TAF qd, 7 of 478 participants (1.5%) acquired HIV, which corresponds to an annualized rate of 1.2 per 100 person-years; 5 were initially assigned to the islatravir qm group and 2 to the FTC/tenofovir disoproxil or FTC/TAF qd group. In participants from the islatravir qm group, the first HIV infection was detected 99 days (range, 99–329 days) after the last dose of islatravir (Figure 2*B*).

## DISCUSSION

This study highlights important progress in long-acting oral PrEP development. While oral and implantable PrEP programs for islatravir have been discontinued, the insights gained are informing the development of the follow-on NRTTI, MK-8527. This study was not conducted as planned due to dose-related decreases in total lymphocytes across the islatravir clinical development program [21, 23]. In this Phase 3 study, the AE profile of islatravir 60 mg qm administered as PrEP was generally similar to that of FTC/tenofovir disoproxil or FTC/TAF qd in cisgender men and transgender women with elevated likelihood of HIV exposure. There were no unexpected events observed in either study group, except for decreases in total lymphocyte counts in the islatravir group. Moreover, no participants acquired HIV during the double-blind phase or within 42 days after discontinuation of either study drug, and no participants from the islatravir qm group acquired HIV within 99 days after the last dose of islatravir. Results of the IMPOWER-24 study are consistent with other studies across the islatravir clinical development program in which the incidence rates of infectious AEs, other than HIV, were similar in participants who received islatravir versus comparators [24, 25]. These findings suggest that the observed reductions in total lymphocyte counts are unlikely to be associated with clinically significant consequences.

Decreases in total lymphocyte counts were observed in participants who received islatravir oral qm and followed a trend of recovery after administration of islatravir was stopped, which was similar to what has been reported previously with islatravir 60 or 120 mg qm and islatravir 20 mg + ulonivirine (MK-8507; 100, 200, or 400 mg qw) [21, 26, 27]. Greater decreases in total lymphocyte counts have been observed at the higher islatravir doses administered qw (20 mg) and qm (60 mg) than with qd treatment with 0.75 mg administration [26]. The decrease in lymphocytes may be caused by accumulation of high islatravir-TP in lymphocytes causing cytotoxicity, possibly through DNA polymerase α inhibition as a contributing factor [21]. A limitation of the current study was that only a small number of participants had total lymphocyte counts measured between screening and study Month 3 of the double-blind phase, because the initial protocol included hematological measurements every 3 months. Moreover, CD4+ T-cell counts were not measured during the first 3 months of the study. However, the high participation rate in the open-label phase, in which hematology values were measured every month, and the long duration of follow-up provide important information about the natural history of variability and of lymphocyte recovery after discontinuation of islatravir. As islatravir concentrations decline in plasma, consistent with its terminal half-life of approximately 195 hours (∼8 days) [14], corresponding improvement in lymphocyte counts are expected.

The original primary efficacy objective could not be assessed because the study was stopped early. It was observed that none of the participants acquired HIV during the double-blind phase nor during the 42-day follow-up. The first acquisition of HIV in a participant who was initially assigned to the islatravir qm group occurred 99 days after discontinuation of islatravir while on open-label FTC/tenofovir disoproxil or FTC/TAF qd.

As a consequence of the dose-dependent lymphocyte decreases seen across the islatravir program, islatravir is not being further developed as oral or implantable PrEP; however, islatravir continues to be investigated at low doses for the treatment of HIV. Another NRTTI, MK-8527, is being evaluated as a potential oral qm PrEP option in 2 Phase 3 studies (NCT07071623; NCT07044297) [28, 29]. MK-8527 has been well tolerated in adults without HIV, and the safety and PK profiles support extended dosing intervals of a week or longer [30]. In adults at low likelihood of HIV exposure, treatment with MK-8527 oral qm did not lead to clinically meaningful changes in total lymphocyte and CD4+ T-cell counts [31]. Preclinical studies with MK-8527 showed no off-target effects in a panel of 114 enzyme/receptor binding assays, which may explain the lack of lymphocyte toxicity [17].

The approved long-acting injectable cabotegravir has demonstrated superior effectiveness compared with FTC/tenofovir disoproxil qd as PrEP [32, 33]. Equally, long-acting injectable lenacapavir, recently approved as twice-yearly self-administered subcutaneous PrEP, has shown superior efficacy in clinical studies when compared with daily oral PrEP or background incidence [34–36]. However, whether long-acting injectable options can be effectively implemented in real-world settings with the same impact seen in clinical studies remains to be determined [37]. The practicalities of cabotegravir implementation raise concerns about the feasibility of injectable products at scale globally [38]. Long-acting oral PrEP, such as MK-8527, which inhibits HIV via the same mechanism as that of islatravir, could provide an alternative choice to overcome barriers associated with low PrEP uptake, adherence, persistence, and implementation [11]. Indeed, long-acting oral PrEP has been observed in some studies to be preferred over other PrEP modalities, including other daily oral and long-acting injectable agents [39]. Although product preferences vary by study and are not always predictive of future clinical decision-making, having more options is likely to meet the needs of a greater number of individuals. Moreover, expanding the range of available PrEP options supports the global efforts to reduce HIV incidence and may facilitate implementation, especially when global resources are increasingly constrained [3].

## CONCLUSIONS

Islatravir 60 mg qm administered as PrEP demonstrated a clinical AE profile generally similar to FTC/tenofovir disoproxil or FTC/TAF qd in cisgender men and transgender women at elevated likelihood of HIV exposure. Consistent with previous studies, decreases in total lymphocyte counts were observed with islatravir but were not associated with an increased rate of infections [26]. Lymphocyte counts showed a trend of recovery over 12 months after islatravir qm was stopped. A novel NRTTI, MK-8527, is currently in clinical development to address the important HIV prevention need for a long-acting oral PrEP agent [17–19].

## Supplementary Material

ciag171_Supplementary_Data

## Supplementary Data

Supplementary materials are available at *Clinical Infectious Diseases* online. Consisting of data provided by the authors to benefit the reader, the posted materials are not copyedited and are the sole responsibility of the authors, so questions or comments should be addressed to the corresponding author.
