# Supplementary material for: Safety and Tolerability of Oral Islatravir Once Monthly as Pre-exposure Prophylaxis in Cisgender Men and Transgender Women Who Have an Elevated Likelihood of HIV-1 Exposure: Results From the IMPOWER-24 Randomized Phase 3 Study
Source: Clin Infect Dis. 2026 Mar 14;83(2):e310–9. doi: 10.1093/cid/ciag171 (PMC13537127; doi:10.1093/cid/ciag171)
Supplement: Supplementary file 1 [file ciag171_supplementary_data.docx]

**Supplementary Materials**

**Table S1**. IMPOWER-24 Sites and Investigators^a^

| Country | Institute | Lead investigator(s) | |
| --- | --- | --- | --- |
| **Brazil** | - **Centro de Referencia e Treinamento DST/AIDS, Sao Paulo** - Instituto Nacional de Infectologia Evandro Chagas, Rio de Janeiro | - **Jose Valdez Ramalho Madruga** - Beatriz Grinsztejn |  |
| **France** | - **Paris Cité University, Paris, France; Assitance Publique - Hôpitaux de Paris, Hôpitaux Saint Louis et Lariboisière, Paris, France and INSERM UMR, Paris, France** | - **Geoffroy Liegeon and Jean-Michel Molina** |  |
| **Japan** | - **Center Hospital of the National Center for Global Health and Medicine, Tokyo** | - **Shinichi Oka and Hiroyuki Gatanaga** |  |
| Kenya | - Nyanza Reproductive Health Society, Kisumu | - Eduard Sanders |  |
| **Peru** | - **Via Libre, Lima** - Asociación Civil Impacta Salud y Educación, Lima | - **Yvett Pinedo** - Javier Lama |  |
| **South** **Africa** | - **Perinatal HIV Research Unit (PHRU)-HIV Prevention CRS, Johannesburg** - **Wits Reproductive Health and HIV Institute (WRHl)-Research Center, Johannesburg** - **Desmond Tutu HIV Foundation, Cape Town, Western Cape** - Josha Research, Bloemfontein | - **Ravindre Panchia** - **Logashnee Govender and Nkosiphile Ndlovu** - **Richard Kaplan** - Johannes Lombaard |  |
| **Thailand** | - **Research Institute for Health Sciences, Chiang Mai** - **Chulalongkorn University, Bangkok** - **HIV Netherlands Australia Thailand Research Collaboration, Bangkok** | - **Khuanchai Supparatpinyo** - **Thanyawee Puthanakit** - **Anchalee Avihingsanon** |  |
| **United** **States** | - **University of Alabama at Birmingham-UAB 1917 Research Clinic, Birmingham, Alabama** - **UCLA Center for Clinical AIDS Research and Education, Los Angeles, CA** - **Global Research Institute, Los Angeles, CA** - **Bridge HIV - San Francisco Department of Public Health, San Francisco, CA** - **The GW Medical Faculty Associates-Medicine, Washington, DC** - **Midway Immunology and Research Center, Fort Pierce, FL** - **University of Miami Miller School of Medicine-Infectious Disease, Miami, FL** - **Orlando Immunology Center, Orlando, FL** - **Ponce De Leon Center Grady Health, Atlanta, GA** - **Howard Brown Health Center, Chicago, IL** - **The University of Mississippi Medical Center, Jackson, MS** - **Rutgers New Jersey Medical School-Clinical Research Center, Newark, NJ** - **The University of North Carolina at Chapel Hill, Chapel Hill, NC** - **Central Texas Clinical Research, Austin, TX** - **The Crofoot Research Center, Houston, TX** | - **Ronnie M. Gravett** - **Raphael J. Landovitz** - **Jay Gladstein and Jorge Rodriguez** - **Susan Buchbinder** - **Marc O. Siegel** - **Moti Ramgopal** - **Susanne Doblecki-Lewis** - **Federico Hinestrosa** - **Igho Ofotokun** - **Catherine Creticos** - **Leandro A. Mena and James B. Brock** - **Shobha Swaminathan** - **A. Lina Rosengren-Hovee** - **Cynthia Brinson** - **Gordon E. Crofoot** |  |

^a^Bold font indicates activated sites for participant enrollment.

**Table S2**. Most Common Infections in All Available Data^a^

| Most common infections (≥1) in any study group, n (%) | Islatravir qm  N = 328 | FTC/tenofovir disoproxil or FTC/TAF qd  N = 166 |
| --- | --- | --- |
| COVID-19 | 107 (32.6) | 58 (34.9) |
| Chlamydial proctitis | 78 (23.8) | 34 (20.5) |
| Gonococcal proctitis | 59 (18.0) | 40 (24.1) |
| Oropharyngeal gonococcal infection | 51 (15.5) | 32 (19.3) |
| Nasopharyngitis | 38 (11.6) | 17 (10.2) |
| Syphilis | 25 (7.6) | 21 (12.7) |
| Chlamydial infection | 17 (5.2) | 9 (5.4) |
| Influenza | 16 (4.9) | 20 (12.0) |
| Pharyngitis | 13 (4.0) | 8 (4.8) |
| Tonsilitis | 12 (3.7) | 5 (3.0) |
| Gastroenteritis | 11 (3.4) | 9 (5.4) |
| Upper respiratory tract infection | 39 (11.9) | 24 (14.5) |
| Bronchitis | 9 (2.7) | 4 (2.4) |
| Gonorrhea | 8 (2.4) | 7 (4.2) |
| Folliculitis | 8 (2.4) | 5 (3.0) |
| Genitourinary chlamydia infection | 5 (1.5) | 7 (4.2) |
| Pharyngitis streptococcal | 4 (1.2) | 7 (4.2) |
| Sinusitis | 4 (1.2) | 6 (3.6) |
| Cellulitis | 4 (1.2) | 2 (1.2) |
| Urethritis gonococcal | 13 (4.0) | 4 (2.4) |

Abbreviations: AE, adverse event; COVID-19, coronavirus disease 2019; FTC, emtricitabine; ISL, islatravir; qd, once daily; qm, once monthly; TAF, tenofovir alafenamide.

^a^Data collected during the double-blind phase and the open-label phase.
